# A Comprehensive Study of the Retinal Phenotype of Rpe65-Deficient Dogs

**DOI:** 10.3390/cells10010115

**Published:** 2021-01-09

**Authors:** Matthew J Annear, Freya M Mowat, Laurence M Occelli, Alexander J Smith, Paul G Curran, James W Bainbridge, Robin R Ali, Simon M Petersen-Jones

**Affiliations:** 1 Department of Small Animal Clinical Sciences, Michigan State University, East Lansing, MI 48824, USA; mattannear@gmail.com (M.J.A.); mowat@wisc.edu (F.M.M.); occelli@msu.edu (L.M.O.); 2 Department of Genetics, UCL Institute of Ophthalmology, 11-43 Bath Street, London EC1V 9EL, UK; alexander.smith@ucl.ac.uk (A.J.S.); j.bainbridge@ucl.ac.uk (J.W.B.); r.ali@ucl.ac.uk (R.R.A.); 3 Center for Statistical Consulting, Michigan State University, East Lansing, MI 48824, USA; curranp1@msu.edu; 4 NIHR Biomedical Research Centre at Moorfields Eye Hospital NHS Foundation Trust and UCL Institute of Ophthalmology, City Road, London EC1V 2PD, UK

**Keywords:** Rpe65, canine, Leber congenital amaurosis, large animal model, ERG, OCT, retinal degeneration

## Abstract

The Rpe65-deficient dog has been important for development of translational therapies of Leber congenital amaurosis type 2 (LCA2). The purpose of this study was to provide a comprehensive report of the natural history of retinal changes in this dog model. Rpe65-deficient dogs from 2 months to 10 years of age were assessed by fundus imaging, electroretinography (ERG) and vision testing (VT). Changes in retinal layer thickness were assessed by optical coherence tomography and on plastic retinal sections. ERG showed marked loss of retinal sensitivity, with amplitudes declining with age. Retinal thinning initially developed in the *area centralis*, with a slower thinning of the outer retina in other areas starting with the inferior retina. VT showed that dogs of all ages performed well in bright light, while at lower light levels they were blind. Retinal pigment epithelial (RPE) inclusions developed and in younger dogs and increased in size with age. The loss of photoreceptors was mirrored by a decline in ERG amplitudes. The slow degeneration meant that sufficient photoreceptors, albeit very desensitized, remained to allow for residual bright light vision in older dogs. This study shows the natural history of the Rpe65-deficient dog model of LCA2.

## 1. Introduction

Mutations in the *Rpe65* gene cause early-onset retinal dystrophies in dogs [1], mice [2,3,4] and humans [5]. In humans, they most commonly cause Leber congenital amaurosis (LCA) type 2 (LCA2), resulting in poor and deteriorating vision starting in childhood. Rpe65 is an isomerase expressed in the retinal pigment epithelium (RPE), where it converts all-*trans* retinyl esters to 11-*cis* retinol. Thus, it plays an essential role in the visual cycle that regenerates and supplies 11-*cis* retinal to the photoreceptors for combination with opsins to form the visual pigments [6,7,8]. Lack of functional Rpe65 disrupts the visual cycle depriving photoreceptors of 11-*cis* retinal, resulting in a marked loss of retinal sensitivity. Residual rod photoreceptor function may result from opsins combining with other retinyl esters such as 9-*cis* retinal that are also present at low levels [9]; and in the case of cones, an additional pathway independent of Rpe65 may allow for some cone chromophore production [10]. However, any such alternative pathway is insufficient to provide normal cone function in the absence of Rpe65 [11]. An important feature of Rpe65 retinopathy is that there is an initial preservation of retinal structure, despite loss of function; a feature that made this dystrophy attractive for gene augmentation therapy (reviewed in [12]). Groundbreaking retinal gene therapy studies in affected dogs (the Rpe65-deficient dog) were the first proof-of-concept studies that led to clinical trials in human subjects for this blinding condition [13]. The Rpe65-deficient dog model was further used in a multitude of studies to refine the therapy and answer questions about treatment at different disease stages and the ability to subsequently treat the contralateral eye without adverse effects from immune responses (reviewed in [14]). Following the completion of a phase III gene augmentation clinical trial for LCA2, the FDA approved voretigene neparvovec (Luxturna, Spark Therapeutics) [15]. This is the first FDA-approved gene therapy for a genetic disease. In addition to being used in the development of gene supplementation therapy, the Rpe65-deficient dog has also been used to test the use of intravitreal retinoids to restore retinal function [16,17].

The mutation underlying the Rpe65 deficiency in dogs occurred in the Briard breed and was initially studied in Sweden [18]. The causal gene mutation was subsequently identified as a 4-basepair frame-shift deletion in *Rpe65* which introduces a premature stop codon, resulting in a lack of functional protein and failure of the visual cycle to produce 11-*cis* retinal [1,19,20]. The original descriptions of the first litter of affected Swedish Briard studied reported that they required “rather bright light to see normally,” whereas dogs produced from crossing a brother and sister from this litter had a more severe impairment of daytime vision, although details of the methodology used to assess vision were not provided [18,21,22]. To allow for an objective, rather than a subjective assessment of canine vision, we developed a four-choice vision testing device [23]. Using this device, we assessed the vision of Rpe65-deficient dogs in our colony. The colony was formed by crossing an affected male Briard with laboratory beagles. The affected dogs were able to successfully identify and exit through the open tunnel of the four-choice device under normal room lighting. However, once that light level was reduced, the dogs were unable to repeatedly identify the open exit tunnel with results showing that they were selecting exit tunnels randomly [23]. Although vision testing has been reported from young dogs, changes in vision with age have not been reported. There is a similar paucity of information on changes in retinal function with age in Rpe65-deficient dogs. Electroretinographic (ERG) assessment of retinal function in younger dogs showed a marked reduction in both rod and cone photoreceptor sensitivity with an elevated dark-adapted threshold of response and reduced response amplitudes, along with severely reduced or absent light-adapted cone flicker responses [20,21]. There are also limited descriptions of the changes in funduscopic appearance that develop with age in Rpe65-deficient dogs. Young animals were reported to have normal appearing fundi [18,19]. Although we identified early retinal thinning in the *area centralis* of affected dogs. This was detectable ophthalmoscopically as hyper-reflectivity from the tapetum in the affected area and was confirmed on optical coherence tomography and by histology [24]. It is reported that older dogs develop multifocal yellow-white spots in the tapetal fundus as well as changes suggestive of retinal thinning and superficial retinal vasculature attenuation [25]. Table S1 lists previous phenotyping studies of Rpe65-deficient dogs.

Morphological studies in younger dogs describe disorientation of the rod outer segment disc membranes from 5 weeks of age, shortening of rod inner segments from 4 months of age, and peripheral rod photoreceptor degeneration from 7 months of age [19,20,22,26]. There appears to be variability in the changes that affect cone photoreceptors between different Rpe65-deficient colonies. Hernández et al. performed an immunohistochemical (IHC) study on eyes from four Rpe65-deficient dogs kept either at the Retinal Disease Studies facility (University of Pennsylvania) or the Baker Institute of Animal Health (Cornell University), both derived from the same source, and found that rod and cone numbers were normal up to 17 months of age (the oldest dog examined) and that there was no mislocalization of cone opsins in the examined eyes [27]. In contrast, our studies showed that there was mislocalization of cone opsins even in the youngest dogs and the older dogs had a profound loss of immunoreactivity for S-cone opsin [28]. In addition to the gradual photoreceptor loss, accumulation of lipid-like inclusions in the RPE are a feature of the retinal dystrophy [22]. There are limited histological descriptions of the changes that develop in older Rpe65-deficient dogs. Loss of photoreceptors is reported to progress more quickly in the peripheral retina [22,29]. Cideciyan et al. describe the onset of photoreceptor degeneration being at 4.9 years of age in the inferior retina while the onset of degeneration in the central retina could be delayed to as late as 7 years of age, whereas Wrigstad et al. reported on a 7-year-old dog that showed a reduction in photoreceptors centrally and an almost complete photoreceptor degeneration peripherally which was accompanied by severe changes of the inner retina [22].

Gene augmentation therapy in human patients has not recapitulated the dramatic improvement in retinal function achieved in Rpe65-deficient dogs. In dogs, a marked improvement in ERG response threshold and amplitudes are achieved as well as a significant improvement in visual function in dim light [20,28,30,31,32,33], whereas the human clinical trials have not yet shown a robust full-field ERG improvement, although many of the patients showed improved visual function as assessed by other techniques [15,34,35,36,37,38,39,40,41]. Species differences in the normal level of Rpe65 function is one suggested explanation for this difference in functional rescue from gene augmentation therapy outcomes between the species, with humans potentially needing a much higher level of activity compared to dogs [42]. Studies in humans and dogs suggested that once retinal degeneration was established that therapy did not halt the process [29,43]. Most studies reported treatment in young dogs with well-preserved photoreceptors. To recapitulate the situation in human subjects who have already lost significant numbers of photoreceptors, older dogs have been treated with gene augmentation therapy. These studies showed that rescue was achievable in older animals [29,33], even if it failed to halt a progressive loss of photoreceptors.

Here we report a more detailed study of the functional and morphological retinal changes that occur with age in the very important Rpe65-deficient dog model.

## 2. Materials and Methods

### 2.1. Animals and Ethics Statement

Dogs used in this study were from a breeding colony maintained at Michigan State University. Table 1 provides details of the dogs used. All dogs were cared for and procedures performed in strict accordance with the guidelines of the Association for Research in Vision and Ophthalmology Statement for the Use of Animals in Ophthalmic and Vision Research and were approved by the Institutional Animal Care and Use Committee of Michigan State University (IACUC approved protocols 05-17-075-00, 05-11-106-00, 05-14-090-00, 05-08-076-00).

### 2.2. Ophthalmoscopic Examination

Complete ophthalmic examination was performed including indirect ophthalmoscopy (Welch Allyn, Skaneateles Falls, NY, USA) and fundus photography (RetCam II, Clarity Medical Systems, Pleasanton, CA, USA).

### 2.3. Vision Testing

Vision testing was performed using a four-choice vision testing device as previously described [23,44]. The device consists of a central box into which the dog is placed and which has four exit tunnels. Only one randomly selected exit tunnel is open at the far end for each run. Two measures were recorded—whether the first exit tunnel entered by the dog was the open tunnel and the time taken to exit the device. Evaluation was performed for each eye individually by placement of an eye mask or opaque contact lens over the contralateral eye. Each eye was tested by 7 repeated trials at 3 different light intensities set at the exit of the tunnels (set to approximately 5.7 × 10^−2^, 2.8 × 10^0^ and 7.5 × 10^2^ lux) as measured by a photometer (IL1700 with an SED033/Y/R Illuminance detector, International Light, Peabody, MA, USA) and results for each eye at each light intensity averaged.

### 2.4. Electroretinography

Electroretinography (ERG) was performed under general anesthesia as previously described using ERGJet lenses (Fabrinal SA, La Chaux-de-Fonds, Switzerland) and platinum subdermal reference (0.5 cm from lateral canthus) and ground electrodes (Grass Instruments, Natus Medical Inc., Pleasanton, CA, USA) [45]. Full-field flash ERGs were recorded using a UTAS-E 3000 electrophysiology unit with a Ganzfeld (LKC Technologies, Gaithersburg, MD, USA) and bandpass filter cut off set at 0.5–500 Hz. Dark-adapted ERG responses were recorded following 1 h of dark adaptation, from a series of stimuli ranging from below threshold to a strong stimulus. Interstimulus intervals were increased from 1 s at low intensities to 360 s at the highest luminance to avoid light adapting the rods. Light-adapted ERGs were recorded following exposure to a standard background light of 30 cd/m^2^ for 10 min. a- and b-wave amplitudes were measured in a standard fashion and stimulus–response curves plotted.

### 2.5. In Vivo Morphology

Spectral domain optical coherence tomography (SD-OCT) (Spectralis. Heidelberg, Germany) was performed as previously described [24] using high-resolution cross sections and volume scans. For the current study, layer thicknesses were measured every 0.5 mm extending in a vertical line from the edge of the optic nerve head to 6.5 mm superiorly and inferiorly, avoiding measurement where major superficial retinal blood vessels were present. The retina thickness (from the internal limiting membrane to the external limiting membrane) as well as the outer retinal thickness (which included the outer nuclear layer and outer plexiform layer) were measured.

### 2.6. Histopathology

Retinal morphology was evaluated on plastic-embedded retinal sections collected and processed as previously described [45]. Sections of the whole posterior eyecup were taken in a superior–inferior orientation, and 3 µm sections were cut through the optic nerve head and stained with hematoxylin and eosin for light microscopic analysis.

Retinal morphology was assessed over the entire length of the retinal sections as previously described [45]. The thickness of retinal layers was measured and number of nuclei in the outer nuclear layer (ONL) counted at 2 locations in the superior retina and 2 locations in the inferior retina 1/3 and 2/3rds of the distance from the optic nerve to the *ora cilliaris retinae*. At each location, ONL cell counts were performed on 2 adjacent 200 µm sections and the numbers averaged.

### 2.7. Statistical Analysis

Vision testing outcomes were analyzed using independent samples *t*-tests to evaluate for differences between Rpe56-deficient dogs and wild-type dogs. To assess for correlation between age and ERG, vision testing and histopathology measures, two-tailed Pearson correlation analysis was performed. The ERG, vision testing and histopathology results were further compared using a one-way ANOVA between different age groups. Post-hoc comparisons were by ANOVA with least significant difference (LSD) to identify the specific time-points that were significantly different. Data were analyzed using SAS Proc Mixed v 9.1.2 (SAS Institute Inc, Cary, NC) and data were considered significant at *p* < 0.05.

## 3. Results

### 3.1. Ophthalmic Findings

**Figure 1:**
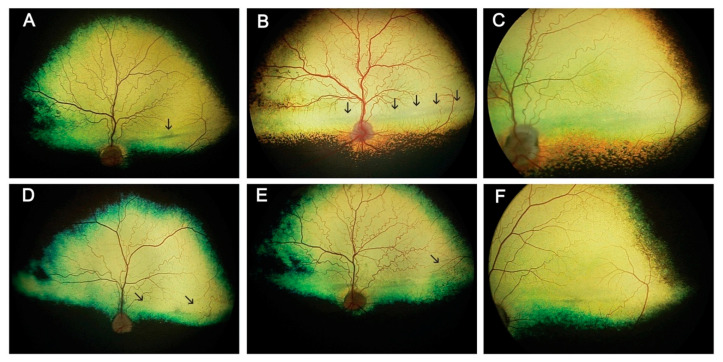
Examples of fundus lesions in the Rpe65-deficient dog. (**A**). Tapetal hyper-reflectivity at the *area centralis* (arrow) 24 months of age. (**B**). Tapetal hypo-reflectivity along the visual streak (arrows) 39 months of age. (**C**). Higher magnification view of the changes in tapetal reflectivity in a dog 47 months of age. (**D**). Dark foci scattered across tapetal fundus (arrows 42 months of age). (**E**). A region of dark foci at the lateral corner of the tapetal fundus (arrows) in a dog 49 months of age. (**F**). Higher magnification view of small pigment foci in the peripheral fundus (56 months of age). The left eye is shown in all images.

The earliest detectable findings were an oscillatory nystagmus and development of a tapetal hyper-reflective lesion in the region of the *area centralis* as previously reported [24]. The *area centralis* lesion was first apparent from as early as 6 weeks of age as a small pinpoint lesion. This finding was accompanied in some dogs by a linear horizontal zone of tapetal hypo-reflectivity in the region of the visual streak [46]. The size of hyper- and hypo-reflective regions increased slowly with age (Figure 1). Tapetal hyper-reflectivity in dogs is seen when there is less than normal attenuation of light passing through the neurosensory retinal and retinal pigment epithelium overlying the reflective tapetum, for example, due to thinning of the retina. Conversely, tapetal hypo-reflectivity occurs when there is increased attenuation of light that reaches and reflects back from the tapetum; indicating retinal thickening or altered transmission of light through the retina.

Other funduscopic lesions detected included the development of small dark-colored foci in the tapetal fundus of the majority of eyes of dogs older than 23 months (Figure 1D–F). Pinpoint hyper-reflective foci scatted across the tapetal fundus developed in three dogs in the older age groups (out of four dogs). Both eyes of two of the three dogs older than 60 months of age had mild attenuation of the superficial retinal vasculature.

### 3.2. Visual Function in Rpe65-Deficient Dogs (Four-Choice Vision Testing Device)

**Figure 2:**
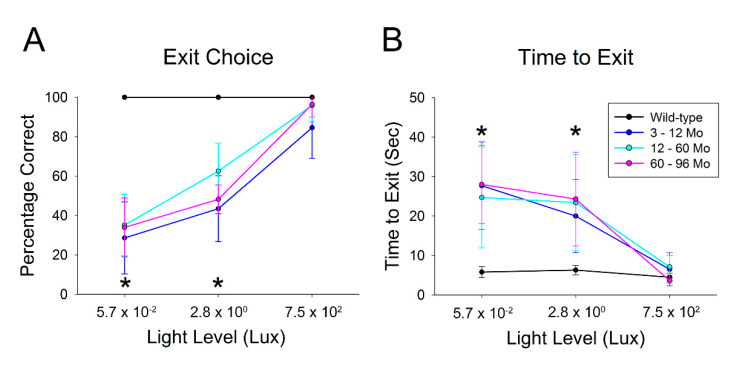
Vision testing results in Rpe65-deficient dogs. (**A**). The mean correct exit choices (± SD) for the wild-type and three age groups of Rpe65-deficient dogs. At full room light (~750 lux), there was no significant difference in correct exit choices. For the intermediate and low light levels (~2.8 and ~0.057 lux), the Rpe65-deficient dogs made significantly more errors in tunnel choice than the control dogs. There were no significant differences in correct exit choices between the three age groups of Rpe65-deficient dogs at any of the light intensities. (**B**). The mean time to exit the vision testing apparatus (± SD). The Rpe65-deficient dogs in all age groups exited the device rapidly and in a similar time to the control dogs. At the lower two lighting levels, they took significantly longer to exit the device than the wild-type controls. There were no significant differences in time to exit between any of the three age groups of Rpe65-deficient dogs at any light intensity. Dogs are grouped as follows: wild-type dogs 6–14 months, *n* = 10 eyes; Rpe65-deficient dogs 3–12 months, *n* = 24 eyes; 12–60 months, *n* = 24 eyes, and 60–96 months, *n* = 8 eyes. * *p* < 0.05.

Rpe65-deficient dogs of each age group tested performed similarly to wild-type control dogs in the four-choice vision testing device under full room lighting levels (approximately 750 lux). There were no significant differences in either outcome measure (exit choice, *p* = 0.14; and time to exit, *p* = 0.68) (Figure 2). At the two lower lighting levels (approximately 2.8 and 0.057 lux), the Rpe65-deficient dogs could not accurately identify the open exit tunnel (at the lowest light level they were randomly selecting exits), whereas the control dogs correctly selected the open exit tunnel at both light levels (the difference was statistically significant *p* < 0.05). The Rpe65-deficient dogs also took significantly longer than the control dogs to exit the device at the two lower light levels (*p* < 0.05). There was no significant difference in either vision testing outcome measure of the Rpe65-deficient dogs between the age groups (*p* value range = 0.10 to 0.75) at any of the test intensities. Note that the four-choice vision testing device is not practical for use in puppies under 3 months of age, so objective assessment of visual function in young puppies was not performed.

### 3.3. Rpe65-Deficient Dogs Have Reduced Retinal Sensitivity with a Decline in ERG Amplitudes with Age

**Figure 3:**
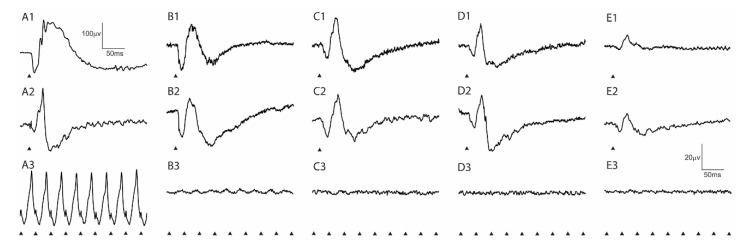
Representative ERG responses showing a decline in amplitudes with age. Shown are representative dark-adapted (1) and light-adapted (2) ERG responses to strong stimuli (2.82 and 2.38 log cdS/m^2^, respectively) and 33 Hz flicker responses (3) (0.39 log cdS/m^2^ imposed on a background light of 30 cd/m^2^). (**A**). Wild-type dog (17 months of age). (**B**–**E**). Rpe65-deficient dogs at 3, 5, 30 and 62 months of age. The triangles indicate the timing of the flash stimulus. Scale: the 20 µv × 50 ms scale bars are for all images except the wild-type dark-adapted recording (**A1**) which uses the 100 µv × 50 ms scale bars.

**Figure 4:**
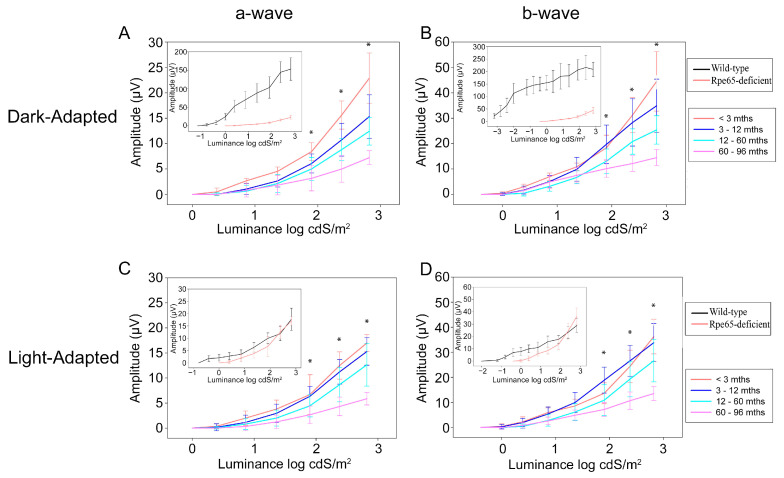
Mean (± SD) ERG amplitude stimulus–response plots in dark- and light-adapted eyes. In each instance, the insert compares the mean wild-type amplitudes of wild-type controls with the 2–3-month-old Rpe65-deficient dogs (note difference in response threshold between wild-type and Rpe65-deficient dogs). (**A**,**B**) Dark-adapted and (**C**,**D**) light-adapted mean a-wave (**A**,**C**) and b-wave (**B**,**D**). Note that amplitudes reduce significantly with age (* indicates flash stimuli with a significant change in amplitude [*p* < 0.05] with age) <3 months, *n* = 8 eyes; 3–12 months, *n* = 34 eyes; 12–60 months, *n* = 28 eyes; 60–96 months, *n* = 8 eyes. Wild-type control dogs: 9–12 months, *n* = 10 eyes.

Representative dark- and light-adapted single-flash ERG tracings in response to strong stimuli and light-adapted cone flicker responses from wild-type control and increasing ages of Rpe65-deficient dogs are shown in Figure 3. The stimulus vs. response functions are plotted in Figure 4 (dark-adapted and light-adapted a- and b-wave amplitudes with results from wild-type control dogs compared to the Rpe65-deficient dogs shown as inserts). The dark-adapted and light-adapted single-flash ERG responses in the Rpe65-deficient dogs were similar in both amplitude and shape and showed similar response thresholds. In contrast, the normal wild-type dog dark-adapted ERG waveform has a broader b-wave than the light-adapted response and much larger a- and b-wave amplitudes and a very different response threshold (Figure 3A and shown in inserts in Figure 4A–D). The dark-adapted b-wave response thresholds of the Rpe65-deficient dogs were elevated over 3 log units while the light-adapted thresholds were elevated by ~1.5 log units. These residual responses decreased in amplitude with age (also shown in Figure 4). The amplitudes of the single-flash light-adapted responses were closer in magnitude to those of the wild-type dogs, although the response threshold was elevated (Figure 4C,D inserts), whereas the cone flicker response were either of very low amplitude or not detectable (Figure 3).

It should be noted that at the standard flash intensity used for light-adapted cone flicker, the single-flash light-adapted response in Rpe65-deficient dogs was also of a very low amplitude.

The mean dark-adapted and light-adapted ERG a- and b-wave amplitudes of the Rpe65-deficient dogs showed a significant decline with age in response to the strongest three flashes used (1.9, 2.4 and 2.8 log cdS/m^2^) (*p* value range = 0.004 to < 0.001; Figure 4). The stimulus strength required to induce a recordable waveform remained similar between the age groups (i.e., the response threshold remained stable but as already stated markedly elevated compared to normal controls).

### 3.4. Slowly Progressive Retinal Thinning and Development of RPE Inclusions

**Figure 5:**
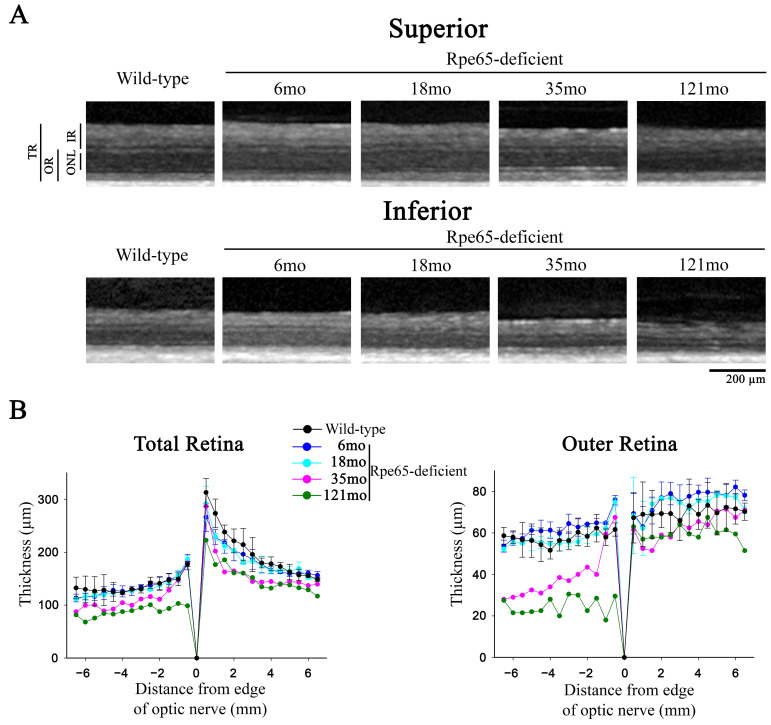
SD-OCT shows that outer retinal thinning with disease progression occurs more rapidly in the inferior retina than the superior retina. (**A**). Representative high-resolution cross-sectional imaging of two retinal regions in wild-type control dog (39-month-old) and 6-, 18-, 35- and 121-month-old Rpe65-deficient dogs at 4 mm superior to the optic nerve head and 4 mm inferior to the optic nerve head. Note the progressive decline in total retinal thickness with age. The thinning is predominantly due to decreases in the outer retinal layers and progresses more rapidly in the inferior retinal region. The difference between the thickness of the outer retinal layers between the inferior and superior retina is pronounced in the 35-month and 121-month-old animals. (**B**). Spidergrams of the total retinal thickness (measured from internal to external limiting membranes) and outer retinal thickness (encompassing ONL to OPL) in a vertical plane through the optic nerve head shown for and 6-, 18-, 35- and 121-month-old Rpe65-deficient dogs (see Table 1) and wild-type control dogs (*n* = 3, mean age 35 months). The total retinal thickness is lower in the inferior retina in all dogs. There is a progressive thinning of the outer retina with age, which is most pronounced in the inferior retina. Key: TR, total retina; OR, outer retina; IR, inner retina; ONL, outer nuclear layer; OPL, outer plexiform layer.

SD-OCT was performed to allow in vivo assessment of retinal layer thicknesses at a range of ages (Figure 5). The inferior retina was thinner than the superior retina in wild-type dogs and all ages of Rpe65-deficient dogs. This difference was predominantly due to a thinner inner retina and the difference in thickness in the outer retina between the two regions was less apparent in the wild-type dogs and younger Rpe65-deficient dogs. There was a decrease in both total retinal thickness and outer retinal thickness with age in the affected dogs apparent at 35 and 121 months of age. This was more pronounced inferiorly and was predominantly due to a marked thinning of the outer retina apparent in the 35 and 121 month ages. The low numbers of dogs at the older ages precluded statistical analysis. Older wild-type control dogs did not show the same degree of thinning with age (data not shown). Detailed analysis of the photoreceptor loss in the *area centralis* of dogs from this colony has been published previously [24].

**Figure 6:**
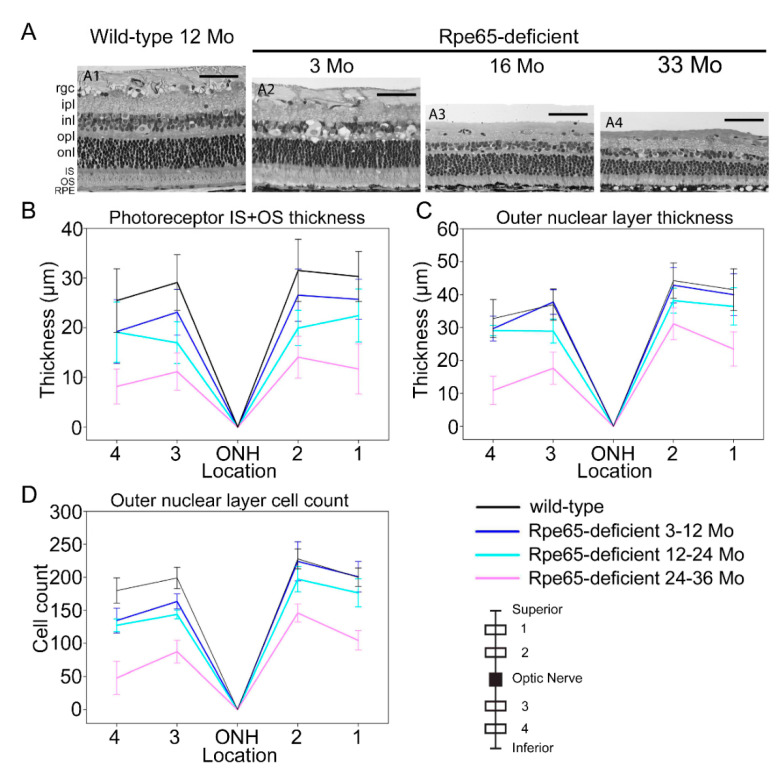
Changes in retinal layer thickness with age in the Rpe65-deficient dog. (**A**). Representative images at site 3 are shown in (**A1**–**A4**); a wild-type control dog 12 months of age (**A1**); and Rpe65-deficient dogs, 3 months of age (**A2**), 16 months of age (**A3**), and 33 months of age (**A4**). (Scale bars = 50 µm.) (**B**). Changes in combined inner/outer segment layer thickness with age. (**C**). Changes in outer nuclear layer thickness with age. (**D**). Changes in outer nuclear layer cell counts with age. Measurements were performed at 4 sites, 1/3 and 2/3 between the optic nerve and *ora ciliaris retinae* both superior and inferior to the optic nerve (see inset schematic). Dogs are grouped as follows: wild-type control dogs 3–36 months, *n* = 11 eyes; Rpe65-deficient dogs 3–12 months, *n* = 7 eyes; Rpe65-deficient dogs 12–24 months, *n* = 5 eyes; and Rpe65-deficient dogs 24–36 months, *n* = 5 eyes.

Detailed assessment of retinal layers on plastic-embedded sections was performed on three groups of younger Rpe65-deficient dogs (3–12, 12–24 and 24–36 months of age) and compared with wild-type dogs within this age range to allow detection of early changes in retinal layer thicknesses. Measurements of retinal layer thicknesses at four sites vertically through the optic nerve head showed that there was a progressive thinning of the retina over this age range (Figure 6 and Figure S1). The thinning was more severe and occurred earlier in the inferior retinal regions, with the outer retinal layers being more severely affected (Figure 6B–D and Figure S1). Over the age range assessed, there was significant thinning of all layers (*p* = 0.02 to 0.001) except the combined nerve fiber/ganglion cell layer (Figure 6B,C and Figure S1). The most notable changes were thinning of the combined inner and outer segment layer (reduced length of inner plus outer segments) (Figure 6B) and thinning of the outer nuclear layer (Figure 6C). This was accompanied by a reduced number of photoreceptor nuclei per unit length (*p* = 0.004) (Figure 6D).

**Figure 7:**
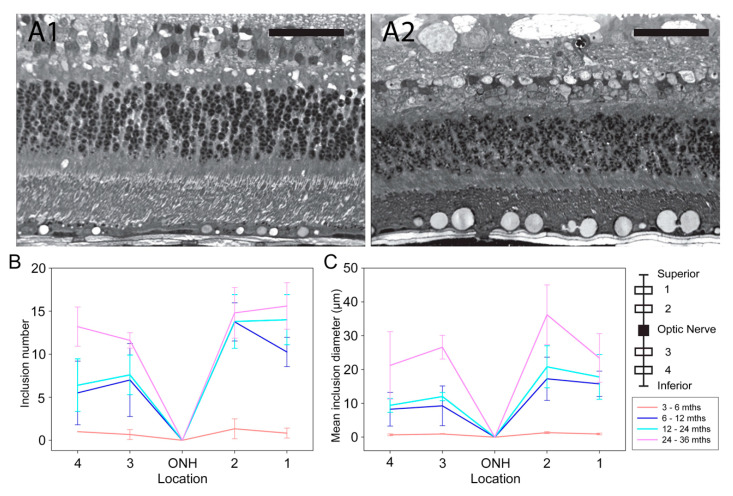
RPE inclusions increase in size and number with age. (**A**). Plastic sections showing RPE inclusions in Rpe65-deficient dogs at 3 (**A1**), and 36 months of age (**A2**). (Scale bars = 50 µm.) (**B**). Changes in the mean number of RPE inclusions with age (number per 200 µm). (**C**). Changes in mean diameter of inclusions (micrometers). Dogs are grouped as follows: 3–6 months, *n* = 3 eyes; 6–12 months, *n* = 4 eyes; 12–24 months, *n* = 5 eyes; 24–36 months, *n* = 5 eyes. Inset shows the sampling positions.

The RPE layer increased in thickness with age (*p* = 0.01) (Figure S1) in the Rpe65-deficient dogs and this appeared to be largely due to the development of RPE inclusions (Figure 7), which were absent in the wild-type controls. The RPE inclusions were sparsely present at 3–6 months (1.0 ± 1.0 inclusions per 200 µm), increasing in number to 13.8 ± 2.6 inclusions per 200 µm at 24–36 months (Figure 7B). A similar increase in the size of inclusions was appreciated as the animals got older, from a mean of 1.0 ± 0.5 µm in cross-sectional diameter at 3–6 months to 27 ± 7 µm at 24–36 months of age (Figure 7C).

## 4. Discussion

The Rpe56-deficient dog has been an important dog model for the development and refinement of gene augmentation therapy. The phenotype of young Rpe65-deficient dogs has been described, often as a prelude to gene therapy trials. However, there is relatively limited information on the progression of the phenotype with age. This paper provides a more detailed longitudinal analysis of the phenotype.

There is some variability in the descriptions of the phenotype of young Rpe65-deficient dogs, most notably with respect to visual function, with some authors describing diminished vision only in dim light, while others report blindness irrespective of lighting conditions [1,18,19,30,32]. Our previous investigations using an objective and quantifiable four-choice vision testing device showed that young Rpe65-deficient dogs have adequate vision in normal room light to accurately and rapidly choose the correct exit choice from the device. However, once the lighting level is reduced to mesopic levels, the dogs become blind paralleling the markedly desensitized photoreceptor function revealed by electroretinography. We now show that vision in bright room lighting (photopic conditions) is maintained up to at least 8 years of age. This is despite a progressive loss of photoreceptors and a decline in the remaining desensitized ERG responses with age. It should be noted that the four-choice vision testing device does not attempt to assess visual acuity.

Consistent with previous studies, young Rpe65-deficient dogs had dark-adapted ERG responses of reduced amplitude with threshold elevated by over 3 log units [13,20,21,30,32], whereas the light-adapted ERG responses of the young dogs were of comparable amplitude to normal dogs but still showed an elevated response threshold [20]. A notable feature of the phenotype is the similarity in amplitude between dark- and light-adapted responses. This may be because the rod suppressing background light routinely used for recording light-adapted ERGs is not adequate to suppress the responses from the desensitized rods in the Rpe56-deficient dog [12]. Under this scenario, the light-adapted ERG waveforms represent the summation of residual cone function responses combined with the response from desensitized rods. The cone flicker responses were either markedly reduced or below detection levels which may reflect a lack of cone function or markedly reduced sensitivity. We observed a slow progressive decline of both light- and dark-adapted a- and b-wave amplitudes as the animals aged, although recordable responses were reliably detected up to 8 years of age. Additionally, despite the declining amplitudes recorded with age, the response thresholds remained relatively stable with age. With respect to cone photoreceptor function, other authors have reported no or very low amplitude cone flicker responses in young dogs [13,21,30]. Our study findings were in accord with this but also showed that these very low amplitude responses were intermittently recorded from dogs of all but the oldest age groups. Similarly to the Rpe65-deficient dog and mouse models, LCA2 patients have severely abnormal ERGs [2,3,47]. In a study of 30 LCA2 patients, of which 29 had ERGs studies, Jacobson et al. reported that an ERG tracing could only be recorded in 59% of the patients studied. When present, the ERG showed that there was a marked loss of retinal sensitivity [47]. It should be noted that the patients in the study were from 4 to 55 years of age when examined, so it is conceivable that some of the older patients could have had recordable ERGs when younger. However, in contrast to LCA2 patients, both the dog (this study) and mouse models have recordable ERG tracings to stronger stimuli which is maintained in older animals (dogs—this study; mice models—[3,48]). The reason for the more severe effects on retinal function, as assessed by the ERG, in human subjects compared to mouse and dog models is not clear. Experimental studies provided evidence that in the mouse models at least, the residual photoreceptor function is due to the generation of low amounts of 9-*cis*-retinal that in the case of rods can combine with opsin to form isorhodopsin [9]. Studies with the *Rpe65^−/−^* and *rd12* mouse utilizing double knockouts where lack of Rpe65 function was combined with either a lack of rod, or a lack of cone function, showed that the residual ERG in both these Rpe65-deficient mouse models was derived from very desensitized rods [49,50]. The mouse rods were desensitized by 3–4 log units, which is very similar to the change in response threshold we show here in the dark-adapted Rpe65-deficient dog model.

Cone function might be expected to be preserved longer than rod function, as alternative pathways, in addition to the classical visual cycle, are recognized for regeneration of retinoid for cones (see [51] for a recent review). It is suggested from psychophysical assessment that the residual vision in human subjects is primarily cone derived, with 41% of patients in one study only having cone-mediated function [47]. However, studies in dog and mouse models suggest that the short-wavelength cones are lost early in animals with Rpe65 deficiency [28,52]. The reason for the maintained presence of desensitized rod function in the dog and mouse models, and yet not in human LCA2 subjects, is not clear. However, the maintenance of low-level rod function rather than complete absence of rod function could explain the relatively slow rod loss and also the dramatic rescue of a relatively normal ERG response that is a feature of gene augmentation therapy particularly in the dog (see ref [14] for a review) and to a lesser extent mouse models [53,54,55,56,57]. In contrast, ERG rescue is not detectable in the majority of LCA2 patients that have undergone gene augmentation therapy (see [12] for a review and [38,42,58]).

With the exception of our previously published study on change in the *area centralis*, there is little published information on the early changes in retinal layer thickness. Here we show by SD-OCT and measurements of plastic sections that there is already thinning of the outer retina by 3 years of age. On histopathology, there was evidence of progressive thinning of all retinal layers with the exception of the RPE and ganglion cell/nerve fiber layer. Changes in the thickness of retinal layers was observed in the youngest dogs in this study. This finding is consistent with some previous studies [22,26] but in contrast with the study of Cideciyan et al. who found that in the retinal regions they assessed photoreceptor degeneration started at 4.9 years of age inferiorly, 5.3 years of age superiorly and even later in the central retina [29]. We also found that the inferior retina was affected earlier and more severely than the superior retina. This is similar to the findings in some LCA2 patients where there is also a more severe retinal thinning in the inferior retina [59].

Some of the difference in timing of outer retinal thinning could be that identical retinal regions were not measured between the studies, but it is more likely that there are real differences between colonies. Although all Rpe65-deficient dogs are homozygous for the same mutation originating from a single founder animal [19], different background genetics and the presence of different potential modifying loci are likely to play a role in the phenotype differences between colonies. Another difference in phenotype between colonies of Rpe65-deficient dogs is the development of progressive retinal thinning at the center of the *area centralis* due to loss of photoreceptors which is similar to macular thinning reported in patients with *Rpe65* mutations [60]. We have reported this as an early feature in our colony [24]. However, until a recent report by Gardiner et al. [43], it had not been described in Rpe65-deficient dogs from other colonies. The recent study from Gardiner et al. reported the feature in older dogs (5 years of age), whereas in our colony it is present from a few months of age [24]. It seems likely that the tight stacking density of photoreceptors (both rods and cones) in the *area centralis* [46] competing for residual retinoid contributes to their early loss. It is likely that there are differences in the density of photoreceptors in the *area centralis* between different dog breeds as has previously been described for retinal ganglion cells [61]. Such a variation could explain the difference in onset of *area centralis* degeneration between colonies as could differences in background genetics.

Rpe65-deficient dogs develop large lipid-like inclusions in the RPE [22,26]. In this study, we have described the dynamics of the development and progression of the inclusions in more detail. We found that multiple small inclusions develop between 6 and 12 months of age and after this age, there is a slow increase in number of inclusions but a substantial increase in their size as more material accumulates.

Taken together, the data presented here provide a detailed and extensive summary description of the phenotype and natural course of retinal functional and structural changes in the Rpe65-deficient dog which is relevant for current and future studies evaluating the outcome of therapeutic interventions in this important model of LCA2.

## Figures and Tables

**Table 1:** Rpe65-deficient dogs and (eyes) used in this study.

Age	Histology	ERG	Vision Assessment	Optical Coherence Tomography	Fundus Appearance
1–3 mths		4(8)		4(8)	4(8)
3–12 mths	5(7)	17(34)	12(24)	5(10)	17(34)
1–2 yrs	3(5)	6(12)	5(10)	2(4)	6(12)
2–3 yrs	4(5)	6(10)	4(8)	3(6)	6(10)
3–4 yrs		2(4)	2(4)	1(2)	2(4)
4–5 yrs		1(2)	1(2)		1(2)
5–6 yrs		2(4)	2(4)	1(2)	2(4)
6–7 yrs					
7–8 yrs		2(4)	2(4)	1(2)	2(4)
10 yrs				1(2)	1(2)
TOTAL	12(17)	40(78)	28(56)	18(36)	40(80)

## Data Availability

The data presented in this study are available on request from the corresponding author.

## Supplementary Materials

The following are available online at https://www.mdpi.com/2073-4409/10/1/115/s1, Figure S1: Changes in retinal layer thicknesses with age and retinal position (measured from plastic-embedded sections). Table S1. Published data on untreated Rpe65-deficient dogs and (eyes).
