# Supplementary material for: A Comprehensive Study of the Retinal Phenotype of Rpe65-Deficient Dogs
Source: Cells. 2021 Jan 9;10(1):115. doi: 10.3390/cells10010115 (PMC7827248; doi:10.3390/cells10010115)
Supplement: Supplementary file 1 [file cells-10-00115-s001.pdf]

Supplemental Materials

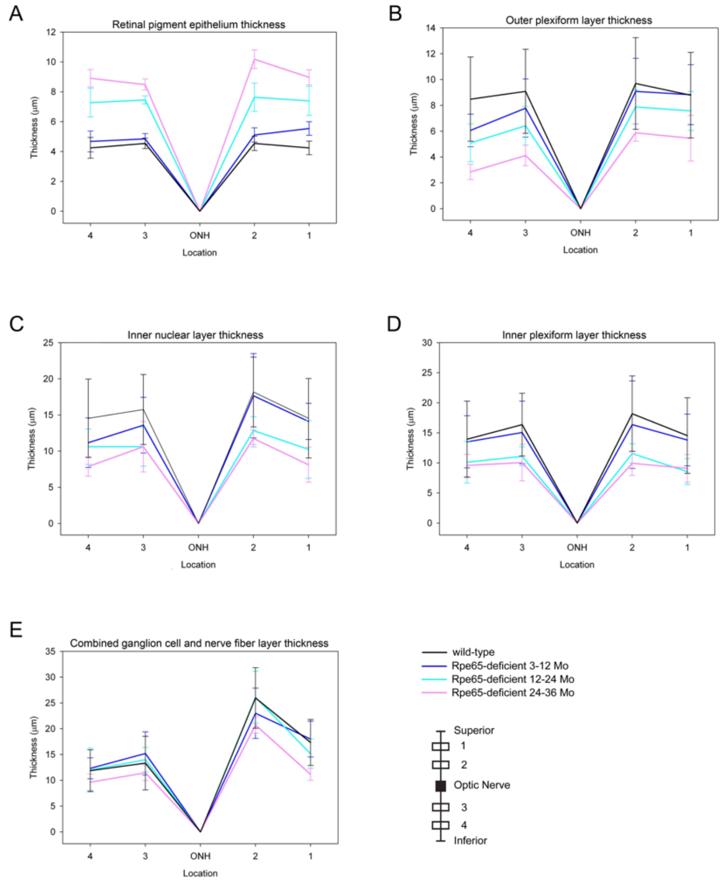

Supplement Figure 1. Changes in retinal layer thicknesses with age and retinal position (measured from plastic embedded sections).
A. RPE; B. Outer plexiform layer; C. Inner nuclear layer; D. Inner plexiform layer; E. Combined ganglion cell and nerve fiber layer. Measurements were performed at 4 sites, 1/3 and 2/3rds between the optic nerve and *ora ciliaris retinae* both superior and inferior to the optic nerve (see inset schematic). Dogs are grouped as follows; wild-type control dogs 3-36 months, n=11 eyes; Rpe65-deficient dogs 3-12 months, n=7 eyes; Rpe65-deficient dogs 12-24 months, n=5 eyes; and Rpe65-deficient dogs 24-36 months, n=5 eyes.

Supplemental Table 1. Published data on untreated Rpe65-deficient dogs and (eyes).[1-13]

Age	Histology	ERG	Vision Assessment	Optical Coherence Tomography	Electron Microscopy	Fundus appearance
1 - 3 mths	1(1)					
3 - 12 mths	12(12)	43(73)	44(64)		5(5)	17(32)
1 - 2 yrs		2(2)	10(10)	9(9)		
2 - 3 yrs	1(1)	1(2)	1(2)			1(2)
3 - 4 yrs		1(1)				
4 - 5 yrs						
5 - 6 yrs						
6 - 7 yrs	1(1)				1(1)	
7 - 8 yrs						
TOTAL	15(15)	47(78)	55(76)	9(9)	6(6)	18(34)

1. Wrigstad, A.; Narfström, K.; Nilsson, S.E. Slowly progressive changes of the retina and retinal pigment epithelium in Briard dogs with hereditary retinal dystrophy. A morphological study. *Doc Ophthalmol* **1994**, *87*, 337-354.
2. Nilsson, S.E.; Wrigstad, A.; Narfström, K. Changes in the DC electroretinogram in Briard dogs with hereditary congenital night blindness and partial day blindness. *Exp Eye Res* **1992**, *54*, 291-296.
3. Acland, G.M.; Aguirre, G.D.; Ray, J.; Zhang, Q.; Aleman, T.S.; Cideciyan, A.V.; Pearce-Kelling, S.E.; Anand, V.; Zeng, Y.; Maguire, A.M., et al. Gene therapy restores vision in a canine model of childhood blindness. *Nat Genet* **2001**, *28*, 92-95.
4. Aguirre, G.K.; Komaromy, A.M.; Cideciyan, A.V.; Brainard, D.H.; Aleman, T.S.; Roman, A.J.; Avants, B.B.; Gee, J.C.; Korczykowski, M.; Hauswirth, W.W., et al. Canine and human visual cortex intact and responsive despite early retinal blindness from RPE65 mutation. *PLoS.Med.* **2007**, *4*, e230.
5. Ford, M.; Bragadottir, R.; Rakoczy, P.E.; Narfstrom, K. Gene transfer in the RPE65 null mutation dog: relationship between construct volume, visual behavior and electroretinographic (ERG) results. *Doc Ophthalmol* **2003**, *107*, 79-86.
6. Narfström, K.; Wrigstad, A.; Nilsson, S.E. The Briard dog: a new animal model of congenital stationary night blindness. *Br J Ophthalmol* **1989**, *73*, 750-756.
7. Narfström, K.; Katz, M.L.; Bragadottir, R.; Seeliger, M.; Boulanger, A.; Redmond, T.M.; Caro, L.; Lai, C.M.; Rakoczy, P.E. Functional and structural recovery of the retina after gene therapy in the RPE65 null mutation dog. *Invest Ophthalmol Vis Sci* **2003**, *44*, 1663-1672.
8. Narfström, K.; Katz, M.L.; Ford, M.; Redmond, T.M.; Rakoczy, E.; Bragadottir, R. In vivo gene therapy in young and adult RPE65-/- dogs produces long-term visual improvement. *J Hered* **2003**, *94*, 31-37.

9. Jacobs, J.B.; Dell'Osso, L.F.; Hertle, R.W.; Acland, G.M.; Bennett, J. Eye movement recordings as an effectiveness indicator of gene therapy in RPE65-deficient canines: implications for the ocular motor system. *Invest Ophthalmol Vis Sci* **2006**, *47*, 2865-2875.
10. Aguirre, G.D.; Baldwin, V.; Pearce-Kelling, S.; Narfström, K.; Ray, K.; Acland, G.M. Congenital stationary night blindness in the dog: common mutation in the RPE65 gene indicates founder effect. *Mol Vis* **1998**, *4*, 23.
11. Acland, G.M.; Aguirre, G.D.; Bennett, J.; Aleman, T.S.; Cideciyan, A.V.; Bennicelli, J.; Dejneka, N.S.; Pearce-Kelling, S.E.; Maguire, A.M.; Palczewski, K., et al. Long-term restoration of rod and cone vision by single dose rAAV-mediated gene transfer to the retina in a canine model of childhood blindness. *Mol Ther* **2005**, *12*, 1072-1082.
12. Wrigstad, A.; Nilsson, S.E.; Narfström, K. Ultrastructural changes of the retina and the retinal pigment epithelium in Briard dogs with hereditary congenital night blindness and partial day blindness. *Exp Eye Res* **1992**, *55*, 805-818.
13. Le Meur, G.; Stieger, K.; Smith, A.J.; Weber, M.; Deschamps, J.Y.; Nivard, D.; Mendes-Madeira, A.; Provost, N.; Pereon, Y.; Cherel, Y., et al. Restoration of vision in RPE65-deficient Briard dogs using an AAV serotype 4 vector that specifically targets the retinal pigmented epithelium. *Gene Ther* **2007**, *14*, 292-303.
